# MARIMO cells harbor a *CALR* mutation but are not dependent on JAK2/STAT5 signaling

**DOI:** 10.1038/leu.2014.285

**Published:** 2014-10-17

**Authors:** K Kollmann, J Nangalia, W Warsch, H Quentmeier, A Bench, E Boyd, M Scott, H G Drexler, A R Green

**Affiliations:** 1 Cambridge Institute for Medical Research, Wellcome Trust/MRC Stem Cell Institute and Department of Haematology, University of Cambridge, Cambridge, UK; 2 Wellcome Trust Sanger Institute, Hinxton, Cambridge, UK; 3 Cambridge University Hospitals NHS Foundation Trust, Cambridge, UK; 4 Leibniz-Institute DSMZ, German Collection of Microorganisms and Cell Cultures, Braunschweig, Germany

Mutations in *calreticulin* (*CALR*) were recently described to be present in the majority of patients with a *JAK2*-unmutated myeloproliferative neoplasm (MPN).^1,2^ This discovery has had rapid clinical impact, and testing for *CALR* has been embedded in national and international diagnostic guidelines.^3, 4, 5^ However, a human MPN-derived cell line harboring a *CALR* mutation has not been reported and the mechanisms by which mutated-*CALR* results in an MPN remain unclear.

To begin to investigate the pathogenetic consequences of mutant CALR, we searched for patient-derived cell lines harboring *CALR* mutations. None were identified by exome sequencing of 1015 cell lines, including 37 derived from hematopoietic neoplasms.^1^ We therefore looked for cell lines derived from patients with leukemic transformation of a preceding MPN. Given that *CALR* and *JAK2* mutations are almost completely mutually exclusive,^1,2,6^ we focused on four such lines known to lack a *JAK2* mutation (MONO-MAC-6, MARIMO, GDM-1 and ELF-153), and also tested a further 52 other predominantly myeloid cell lines (Supplementary Table 1). Mutation screening was done by Sanger sequencing as previously described,^1^ and details of other methods are in the Supplementary Information.

The only cell line found to harbor a *CALR* mutation was MARIMO, originally derived from a 68-year-old female with AML-M2, and an antecedent history of ET.^7^ MARIMO is negative for *JAK2V617F* and *MPL* exon 10 mutations (data not shown) and carries a heterozygous 61-basepair (bp) deletion in *CALR* exon 9 (c.1099_1159del; L367fs*43), which, like all other reported *CALR* mutations, results in a +1- bp shift in the reading frame and thus generates a novel C terminus (Figure 1a). In patients, the commonest two *CALR* mutations, accounting for 85% of cases, are a 52-bp deletion (type 1; c.1099_1150del; L367fs*46) and a 5-bp insertion (type 2; c.1154_1155_ins; K385fs*47).^8^ Both the type 1 deletion and the MARIMO deletion are immediately preceded by a nucleotide sequence identical to that at the 3' end of the deletion (Figure 1a). The 61-bp MARIMO deletion is readily detected by fragment analysis and represents a useful positive control for diagnostic clinical testing (Figure 1b).

Allele-specific PCR demonstrated expression of the mutant *CALR* allele (Figure 1c). Compared with other cell lines derived from patients with *JAK2V617F* (HEL, UKE-1 and SET-2) or CML (K562) total *CALR* mRNA levels were 10-fold higher in MARIMO (Figure 1d) and total CALR protein levels were also increased albeit more modestly (Figure 1e).

MARIMO cells expressed cell surface marker CD15 but not other progenitor or lineage-affiliated markers (Supplementary Table 2). The proliferation and cell cycle status of MARIMO was unremarkable compared with other myeloid lines (Supplementary Figure 1). We next analysed cellular calcium stores since CALR has an important role in endoplasmic reticulum (ER) mediated calcium homeostasis^9^ and mutant CALR protein lacks variable numbers of calcium binding sites present in the wild-type C terminus. No significant differences in basal cytoplasmic calcium levels were found amongst the six cell lines tested (Figure 2a). Cell lines were then treated with 1 μM thapsigargin, which blocks ER Ca^2+^-ATPase channels resulting in ER calcium depletion and increased cytosolic calcium levels.^10^ MARIMO cells showed the slowest rate of increase of cytoplasmic calcium levels upon addition of thapsigargin (Figure 2b), consistent with the concept that mutant CALR alters ER dependent calcium homeostasis.

The mutual exclusivity of *JAK2* and *CALR* mutations argues that they may share pathogenetic mechanisms and has been used to suggest that *CALR* mutations may activate JAK2/STAT5 signaling. This concept is supported by expression profiling of patient-derived granulocytes^11^ together with a report that expression of CALR in Ba/F3 cells confers interleukin-3 independence and is accompanied by increased STAT5 phosphorylation.^2^ However other studies have reported distinct transcriptional signatures in *JAK2V617F-*mutated and *JAK2V617F-*unmutated MPNs.^12,13^ Interpretation of these apparently conflicting results is complicated by several issues including limitations of overexpression systems, the uncertain relevance of granulocytes to disease pathogenesis and difficulties inherent to studies of signaling in primary cells containing variable proportions of mutant cells. To circumvent some of these issues, and to gain insight into the consequences of *CALR* mutations, we explored the properties of MARIMO cells.

The dependence of MARIMO cells on JAK signaling was initially assessed using the JAK inhibitors Tofacitinib (a JAK2/3 inhibitor) and JAK-inhibitor-I (a pan-JAK inhibitor) (Supplementary Figure 2). MARIMO cells were more resistant to both inhibitors than seven cell lines harboring mutant *JAK2* or *JAK3*. Dose response studies using the clinically approved JAK-inhibitor Ruxolitinib (INCB018424, a JAK1/2 inhibitor) showed that HEL and UKE-1 (both JAK2V617F positive) had IC_50_ values of 217 and 430 nm, respectively (Figure 2c). In marked contrast the IC_50_ value for MARIMO was greater than 10 000 nm, demonstrating that MARIMO was not dependent on JAK2 signaling. Consistent with these data, western blot analysis showed that, compared with *JAK2*-mutant cells, MARIMO cells contained markedly reduced levels of JAK2, phosphorylated-JAK2 (pJAK2), STAT5 and pSTAT5 (Figure 2d). The lack of JAK2-STAT5 signaling was not accompanied by a compensatory increase in STAT1 or STAT3 phosphorylation (Figure 2e). *JAK2* transcript levels in MARIMO were similar to other cell lines (Figure 2f), suggesting either decreased translation or increased degradation of JAK2.

Together, our data demonstrate that the MARIMO cell line harbors a CALR mutation and yet is not dependent on JAK/STAT signaling, in marked contrast to *JAK2*-mutated cell lines. Our results therefore raise the possibility that mutations of *CALR* and *JAK2* may share activation of pathways other than the STATs. Superficially our data appear to contrast with reports that *JAK2*-unmutated and *CALR*-mutated MF patients respond to ruxolitinib.^14,15^ However in JAK2V617F-positive patients studies of mutant allele burden show that Ruxolitinib has a minimal effect on the mutant clone.^15^ It is therefore likely that the clinical responses to Ruxolitinib (reduced splenomegaly and improved constitutional symptoms) do not reflect a cytotoxic effect of the drug on the neoplastic clone, but instead are at least in part due to down-modulation of pro-inflammatory signaling cascades.^16^

## Figures and Tables

**Figure 1:**
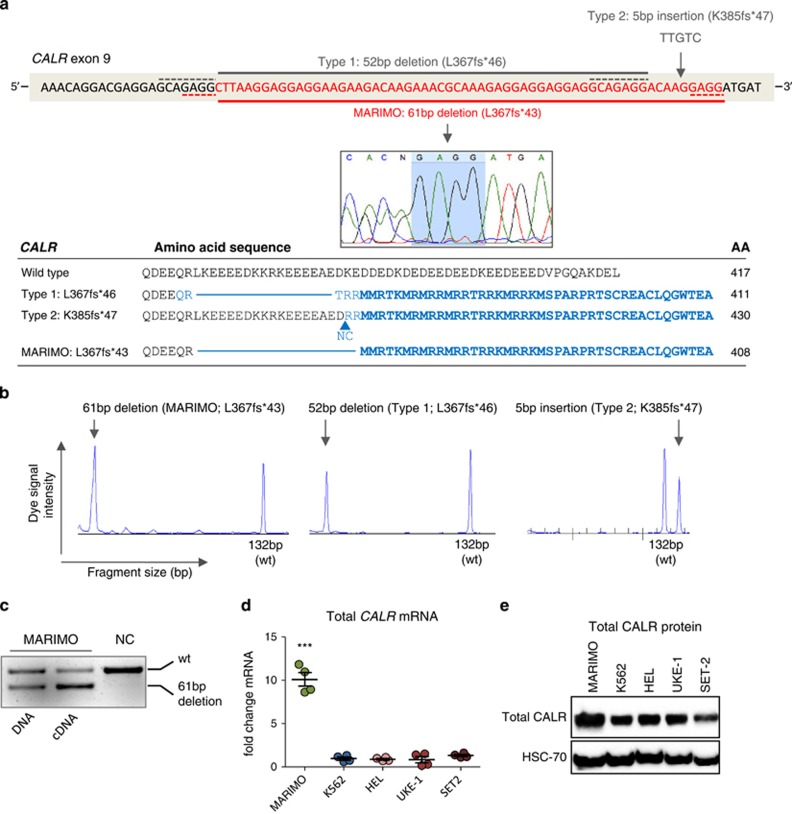
Identification of a *CALR*-mutated human cell line. (**a**) Top panel shows the mutated region in *CALR* exon 9 (red bases). The commonest *CALR* mutations are shown above the DNA sequence. Solid gray line shows type 1 (52- bp deletion; c.1099_1150del; L367fs*46) and gray arrow shows type 2 (5- bp insertion; c.1154_1155_ins; K385fs*47 mutations). The *CALR* mutation in human cell line MARIMO is shown below the DNA sequence. Solid red line and capillary sequencing image show a heterozygous 61-bp deletion (c.1099_1159del; L367fs*43) in MARIMO. Dashed gray and red lines represent the homologous sequence flanking the deleted regions in type 1 and MARIMO mutations, respectively, also highlighted in the capillary sequencing image (pale blue) for MARIMO. Lower panel shows the predicted protein sequence of the commonest *CALR* mutations and of MARIMO with total protein sizes. Amino acids (AA) in the new reading frame are shaded blue and the common novel peptide sequence shared by the different CALR variants are in bold blue. (**b**) PCR amplification of *CALR* exon 9 followed by fragment size analysis, as used for diagnostic testing for *CALR* mutations. Vertical heights of peaks represent dye signal intensity and horizontal position of peaks reflect the fragment size of the PCR amplicon. Wild type (wt) peak occurs at 132- bp. Left panel shows wt and mutated alleles of MARIMO (61-bp separation in peaks), middle panel shows Type 1/L367fs*46 with peak separation of 52 bp and right panel shows Type 2/K385fs*47 peaks separated by 5  bp. (**c**) Agarose gel image showing wt (upper band) and mutated-*CALR* (lower band) in MARIMO DNA and cDNA. (**d**) Quantitative real-time PCR of total *CALR* mRNA levels expressed as a fold change relative to house-keeping *RPLP0* levels, for the cell lines MARIMO, the *BCR-ABL1*^*+*^ CML cell line K562, and the *JAK2V617F*^*+*^ cell lines HEL, UKE-1 and SET-2. Graph depicts all data points generated in two independent experiments performed in duplicate. ***P<0.001 (**e**) Western blot showing total CALR protein levels of MARIMO and four other myeloid cell lines.

**Figure 2:**
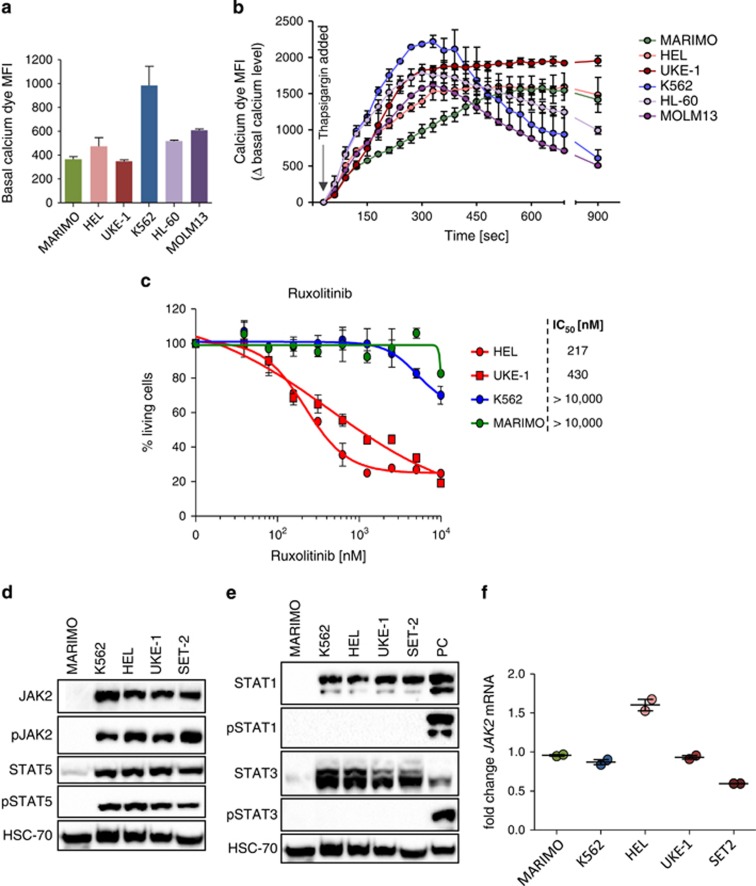
Characterization of the cell line MARIMO. (**a** and **b**) Basal cytoplasmic calcium level (**a**) and changes in cytoplasmic calcium levels over time upon addition of thapsigargin (**b**) in MARIMO and five other leukemic cell lines. (**c**) Dose response curves for the JAK2-inhibitor Ruxolitinib in the cell lines MARIMO, K562, HEL and UKE-1. (**d** and **e**) Western blots showing protein levels of the inactive and phosphorylated forms of JAK2 and STAT5 (**d**) and STAT1 and STAT3 (**e**). PC, positive control. (**f**) *JAK2* mRNA levels expressed as fold changes relative to *RPLP0* in MARIMO and four myeloid cell lines.
