# Supplementary material for: MARIMO cells harbor a CALR mutation but are not dependent on JAK2/STAT5 signaling
Source: Leukemia. 2014 Oct 17;29(2):494–7. doi: 10.1038/leu.2014.285 (PMC4320290; doi:10.1038/leu.2014.285)
Supplement: Supplementary Information [file leu2014285x1.pdf]

SUPPLEMENTARY INFORMATION

SUPPLEMENTARY LEGENDS

Supp. Figure 1. Cell count (**a**) and cell cycle profile (**b**) of MARIMO and control cell lines.

Supp. Figure 2. Relative percentage living cells following 72 hours treatment with the JAK inhibitors Tofacitinib [4 µM] (**a**) and JAK inhibitor I [4 µM] (**b**) of MARIMO (green), JAK-dependent (red) and JAK-independent (blue and violet) cell lines.

Supp. Table 1. List of human hematopoietic cell lines screened for mutations in *CALR.*

Supp. Table 2. Expression of progenitor or lineage-affiliated surface markers on MARIMO and three other myeloid human cell lines.

EXPERIMENTAL PROCEDURES

Cell line screen

We tested acute myeloid leukemia-derived cell lines established from patients with an antecedent history of MPN and known to be *JAK2V617F*-unmutated: MONO-MAC-6 (myeloid metaplasia -> AML M5), MARIMO (ET -> AML M2), GDM-1 (MPN -> AML M4) and ELF-153 (MF -> AML M7), and a further 52 hematopoietic cell lines (Table S1).

Fragment analysis of *CALR* exon 9

2 ng DNA was amplified by PCR in a final volume of 20 µL containing 1 X PCR Buffer II, 1.5 mM $MgCl_2$, 200 µM each dNTP, 0.5 µM each primer ([D4-PA]-CAGCAGAGAAACAAATGAAGGACA and CCTCATCCTCATCTTTGTCCTCATCA) with 1 unit AmpliTaq Gold (Life Technologies, Paisley, UK). PCR conditions were 94°C for 7 min followed by 27 cycles of 94°C for 30 s, 60°C for 30 s and 72 ^{o}C for 30 s with a final extension of 72 ^{o}C for 30 min. PCR products were analysed by mixing 1 µL with 39 µL Sample Loading Solution/DNA size standard – 400 (AB Sciex Ltd, Warrington, UK) and loading onto a CEQ8000 genetic analysis system (Beckman Coulter, High Wycombe, UK). Run duration was 35 minutes with a separation voltage of 6.0 kV. Wild type alleles gave a PCR product of 132 base pairs.

Cell lines and culture conditions

MARIMO, K562, HEL, HL-60, MOLM13, SET-2, Mac2A, CMK, Ku812, LAMA84, KCL-22, MV4;11, Ba/F3-J2V/F and 32D-J2V/F cells were cultured in RPMI (Sigma), 10% FCS (Life Technologies), penicillin/streptomycin (100U/mL, 100mg/mL). UKE-1 cells were cultured in 20% FCS.

Protein lysis and Western Blots

Cell lysates and immunoblotting was performed as described previously.[1] Used antibodies: CALR (Millipore), HSC-70, STAT5 (Santa Cruz), Jak2, pJak2, pSTAT5, STAT1, pSTAT1, STAT3, pSTAT3 (cell signaling).

PCR and real-time PCR

RNA was isolated using TriZol (Invitrogen). First-strand cDNA-synthesis and PCR-amplification were performed using the Tetro cDNA FAST qPCR Master Mix (Bioline) according to the manufacturer's instructions. qPCR was performed using KAPA SYBR FAST qPCR Master Mix (KAPA Biosystem). The following primer sequences were used: *CALR* F 5' GAGCCTGCCGTCTACTTCA 3' and R 5' AACTGAGAACG AATTTGCCA 3', *JAK2* F 5' AAGCTTTCTCACAAGCATTTGGTTT and R 5' AGAAAGGCATTAGAAAGCCTGTAGTT *RPLP0* F 5' GGCGACCTGGAAGTCCAACT 3' and R 5' CCATCAGCACCACAGCCTTC 3'. Each experiment was performed in duplicate and results normalized by comparison to *RPLP0* mRNA expression.

Proliferation and cell cycle assay

Assays were performed as described previously.[1]

Dose response assay

Kinase inhibitors used during the study were purchased from Selleckchem (Tofacitinib, Ruxolitinib) and Calbiochem (JAK inhibitor I) and dissolved in DMSO. Cells were seeded into 96 well plates (100 µl/well, 2 x 10^5 cells/ml) with clear bottom (Greiner bio-one) and treated with inhibitors. To measure the amount of living cells after 72 hours an ATP-based cell-viability assay was performed. CellTiter-Glo (Promega) was

diluted 1:5 in sterile H_2O, 100 µl/well were added to the cells and incubated at RT for 20 minutes. Cell viability was measured in a luminometer (GloMax@ 96, Promega) with an integration time of 1 sec/well. The “% living cells” has been calculated as 100 divided by the average relative luminescence unit (RLU) of untreated control cells multiplied by the RLU of inhibitor treated cells. IC_{50} values have been calculated using GraphPad Prism software.

Calcium release assay

To reduce differences in calcium levels between cell lines caused by non-biological variances (e.g. staining procedure, culture conditions) we barcoded each cell line and performed the experiment in a single FACS tube to enable equal conditions. Each cell line was stained with a distinct concentration (0, 0.1, 0.3, 1, 3, and10 µM) of the fluorescent dye CellTrace Violet (life technologies). The staining reaction was stopped after 20 minutes by adding 10 ml RPMI + 5% FBS to the cells for 10 minutes. All cell lines were washed once with PBS, pooled into a single FACS tube and incubated under normal cell culture conditions for 2 hours. Each cell line is then represented by a distinct population in the violet spectrum of the FACS.

Cells were stained with 6 µM of the calcium dye “eFluor™ 514 Calcium Sensor Dye” (eBioscience) for 30 minutes at 37C and analysed by FACS. After 30 seconds the recording of the cells was paused, Thapsigargin (1 µM endconcentration) was added, cells were shortly vortexed and recorded for another 8.5 minutes.

References:

1. Kollmann, K. et al. A kinase-independent function of CDK6 links the cell cycle to tumor angiogenesis. *Cancer cell* **24**, 167-181 (2013).

Supplementary Figure 1

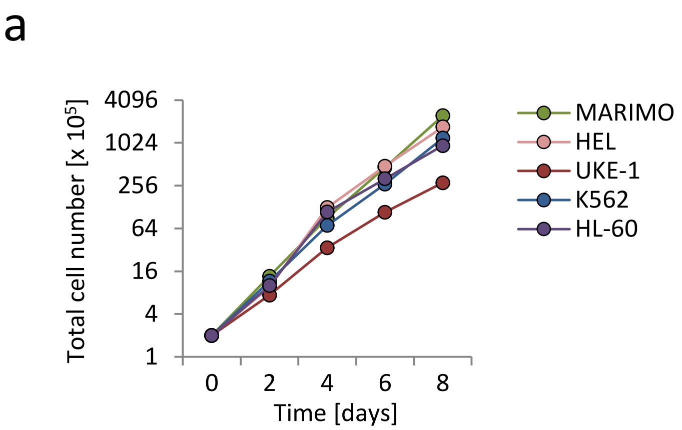

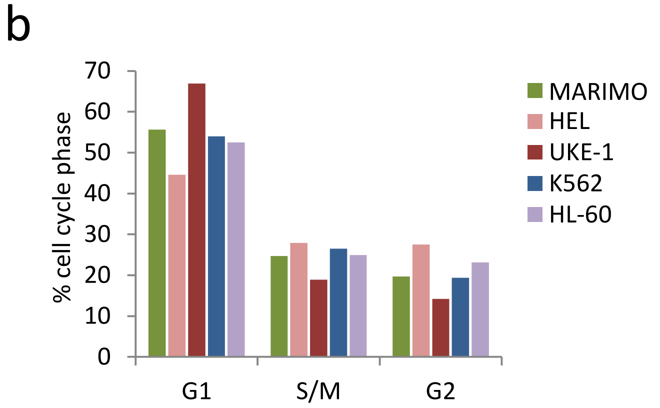

Supplementary Figure 2

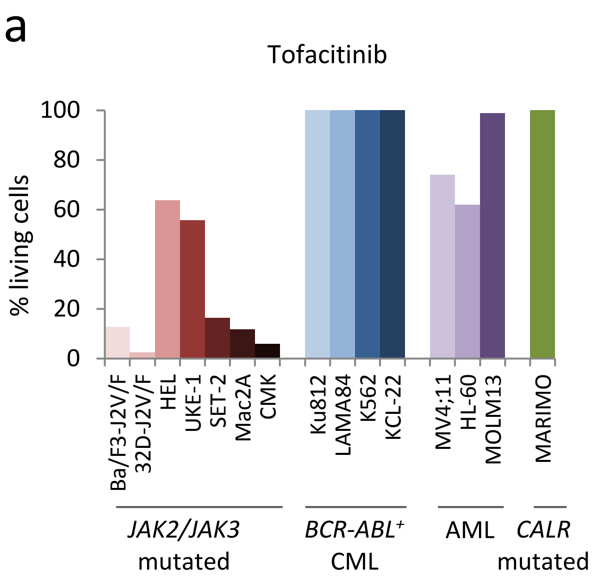

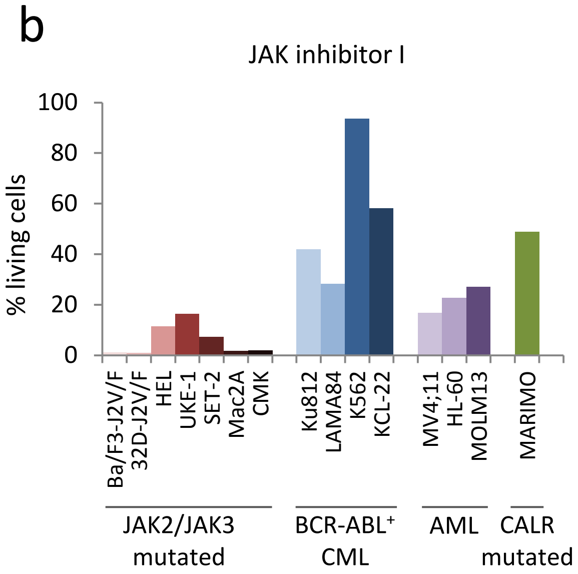

Supplementary Table 1

Cell line screen					
AML-193	GF-D8	M-07e	MONO-MAC-6	OCI-AML3	SKNO-1
AP-1060	HEL	MARIMO	MTT-95	OCI-AML4	TF-1
AS-E2	HL-60	MB-02	MUTZ-3	OCI-AML5	THP-1
CESS	HNT-34	ME-1	MUTZ-8	OCI-M1	U-937
CMK	HU-3	MEGAL	MV4;11	OCI-M2	UCSD-AML1
CTV-1	KASUMI-1	MKPL-1	NB4	PL-21	UKE-1
ELF-153	KASUMI-3	ML-2	NKM-1	QIMR-WILL	
F-36P	KG-1	MOLM-13	NOMO-1	SET-2	
FKH-1	KMOE-2	MOLM-16	OCI-AML1	SIG-M5	
GDM-1	KO52	MOLM-17	OCI-AML2	SKM-1	

Supplementary Table 2

lineage specificity	surface marker	K562	HEL	UKE-1	MARIMO
Megakaryocytic lineage	CD61	+	++	-	-
Megakaryocytic lineage	CD41	-	++	-	-
Megakaryocytic lineage	CD42a	-	+	-	-
Hematopoietic lineage	CD45	++	+	++	-
Erythroid lineage	GPA (CD235a)	++	++	++	-
Progenitor cells	ckit (CD117)	-	+	-	-
Progenitor cells	CD34	-	-	-	-
B-cell lineage	CD19	-	-	-	-
Granulocytic lineage	CD15	+	-	+	+
Monocytic lineage	CD14	++	-	+	-
